# Ukrainian and Russian in the lexicon of Ukrainian Suržyk: reduced variation and stabilisation in central Ukraine and on the Black Sea coast

**DOI:** 10.1007/s11185-023-09286-9

**Published:** 2023-12-18

**Authors:** Gerd Hentschel

**Affiliations:** Carl von Ossietzky University, Oldenburg, Germany

**Keywords:** Suržyk, code-mixing, fused lects, Ukrainian-Russian language contact, quantitative corpus linguistics, Суржик, смешение кодов, слитые лекты (“fused lects”), украинско-русский языковой контакт, количественная корпусная лингвистика

## Abstract

The subject of this study is the so-called “Surzhyk”, a mixed Ukrainian-Russian variety used by millions of people in Ukraine, sometimes alongside Ukrainian and, less commonly, alongside Russian. More specifically, the focus here is on the lexicon, addressing the following questions: (i) To what extent is the mixed speech lexicon influenced by Ukrainian or Russian? (ii) Does the distribution of Ukrainian or Russian lexemes reveal a reduction in variation, i.e. patterns of stabilisation? In other words, are there tendencies for one of the two competing, synonymous, or functionally equivalent Ukrainian or Russian lexemes to prevail over the other?

Many Ukrainian linguists have stereotypically claimed for years that the distribution of Ukrainian and Russian elements in Surzhyk is unpredictable, spontaneous, if not chaotic. It is worth noting that these opinions are not based on comprehensive, systematic empirical evidence and largely ignore theoretical developments in the field of code-mixing.

In contrast, by means of a quantitative analysis of an extensive corpus and a focus on intra-sentential code-mixing, this study demonstrates that the majority of recorded lexical Ukrainian-Russian competitions exhibit a clear fixation on one of the two expressions, resulting in a reduction in variation. In these instances, one of the two expressions prevails extensively across the entire region of Central Ukraine and the Black Sea Coast. Surzhyk is evidently evolving towards a “fused lect”. A smaller portion of the examined instances reveals such stabilisation only in certain parts of the survey area, and another equally small portion exhibits widespread variability. In general, Ukrainian and Russian lexemes are roughly balanced in quantity.

## Аннотация

Предметом данного исследования является так называемый «суржик», смешанный украинско-русский идиом, используемый миллионами людей в Украине, иногда наряду с украинским, реже — с русским. Основное внимание здесь уделяется лексике, при этом рассматриваются следующие вопросы: (i) В какой степени лексика смешанной речи находится под влиянием украинского или русского языка? (ii) Выявляет ли распространение украинских или русских лексем снижение вариативности, т.е. признаки стабилизации? Иными словами, существуют ли тенденции к преобладанию одной из двух конкурирующих, синонимичных или функционально эквивалентных украинской или русской лексем над другой?

Многие украинские лингвисты в течение многих лет стереотипно утверждали, что распределение украинских и русских элементов в суржике непредсказуемо, спонтанно, если не сказать хаотично. Стоит отметить, что эти мнения не основаны на всеобъемлющих, систематических эмпирических данных и во многом игнорируют теоретические разработки в области смешивания кодов.

Напротив, посредством количественного анализа обширного корпуса и акцента на смешение кодов внутри предложений это исследование демонстрирует, что большинство зафиксированных лексических украинско-русских конкурирующих форм проявляет явную фиксацию на одном из двух выражений, что приводит к уменьшению вариаций. В этих случаях одно из двух выражений широко преобладает во всем регионе Центральной Украины и Причерноморья. Суржик явно эволюционирует в сторону “fused lect”. Меньшая часть рассмотренных единиц обнаруживает такую стабилизацию лишь в отдельных частях исследованного региона, а другая, столь же небольшая часть, демонстрирует широкую вариативность. В целом украинские и русские лексемы примерно сбалансированы по количеству.

## Introduction

The lexicon is generally considered the most open subsystem in languages and is that with the lowest degree of stringent structuring on the macro-level.^1^ The lexicon is also generally the area that most rapidly reflects social, technical and cultural change. In terms of language contact, this means that influences of one language on another are most easily reflected in the lexicon. However, the lexicon is in this respect not homogeneous. Thus, the “semantic” lexicon, primarily nouns but also adjectives, certain adverbs and verbs, is more susceptible to external influences than the “functional”, more grammatical lexicon. However, even within these two broad lexical classes, differences still exist. For example, the vast majority of lexical borrowings in all borrowing constellations involves nouns. It should be noted that these observations all relate to type frequency or frequency within the system, and not to token frequency, which is usage frequency.

These universal phenomena are discussed in contact linguistics under the term “borrowing hierarchies/scales” (Matras, 2009, pp. 153–165). Such hierarchies are not only relevant for loan relationships between two (established) languages, but also for contact varieties or contact languages, i.e., more or less stable codes that emerge due to long-term, extensive and intensive contact between languages or dialects.

In the Russian Empire and the Soviet Union (except for a longer period in the 1920s), Russian politically and socially dominated a large number of other languages, including the other East Slavic languages, Ukrainian and Belarusian. Among the speakers of the latter two languages, a form of mixed Ukrainian-Russian or Belarusian-Russian speech spread over many decades, which have been termed “Suržyk” and “Trasjanka”.^2^ Due to the strong structural affinity between the three East Slavic languages,^3^ code-mixing in Muysken’s (2000) typology can largely be described as of the type “congruent lexicalization”. Insertional or alternating code-mixing plays a subordinate role (cf. Tesch, 2014 for Trasjanka). In an early study based on informal but certainly broad observation, Cychun (1998) argued that Trasjanka had been almost completely Russified in the lexicon. Hentschel (2013) tends to confirm Cychun’s rather general observations, but specifies that a comprehensive Russification of the lexicon can only be affirmed for the areas of “semantic lexicon” and possibly “pragmatic lexicon” (e.g. discourse markers, all with exceptions), while in the “functional” domain, various Russian elements have been able to prevail over their Belarusian translation equivalents, but in other cases, the opposite is true, and the Belarusian elements remain firmly established.

Belarusian Trasjanka and Ukrainian Suržyk are certainly comparable phenomena from a historical and sociolinguistic perspective. Both are attributable to the aforementioned long-term dominance of Russian, which was more pronounced in urban than in rural areas. Phenomena like industrialisation and the associated urbanisation or rural-urban migration were relevant prerequisites for the emergence of Suržyk and Trasjanka (Taranenko, 2007; Zaprudski, 2007). However, there are also significant differences between Ukraine and Belarus, both historically and sociohistorically. The Belarusian linguistic area had been under Russian rule since the late 18th century, while the western part of Ukrainian only came under Russian rule after 1945, the area east of the Dnipro River since the second half of the 17th century, and the central region, similar to the previously non-Slavic inhabited Black Sea Coast, only since the late 18th century. This is reflected linguistically in the much stronger position of Ukrainian in the west and of Russian in the east and south (Black Sea Coast). However, it cannot be said that the country is linguistically divided into a Ukrainian and a Russian speaking part, as is sometimes suggested in the media (see recently Hentschel & Taranenko, 2021).

In an initial comparison of Suržyk and Trasjanka, Hentschel (2018) demonstrated the much higher degree of Russification in Trasjanka, which is systematically linked to some of the aforementioned hierarchies. This study aims to present a more differentiated analysis of the Suržyk lexicon, focusing – for methodological reasons (see below) – on frequently-used lexemes.

The central questions for this study are:

(i) To what extent do the findings of the analysis indicate a stabilised mixture or spontaneous mixing? In other words, is the use of competing Ukrainian and Russian lexemes really chaotic, as many Ukrainian colleagues believe?
(ii) To what extent are regional differences recognisable?
(iii) To what extent is the Suržyk lexicon coined by Ukrainian or by Russian, even beyond the units analysed in more detail in this study?

## Short notes on the data and on two possible subtypes of Suržyk

The following analyses of Suržyk are corpus-based. The corpus material was collected in three research projects on Suržyk in central parts of Ukraine (eleven oblasts) and in another project on the Ukrainian Black Sea Coast (three oblasts).^4^ The corpora contain approximately 730,000 word forms in total. About 47 percent of these stem from the central region, and 53 percent from the south. The average corpus size per oblast is significantly larger in the south. This is mainly because the project in the three southern oblasts of Odesa, Mykolaïv and Xerson aims to investigate empirical evidence for a Russian-based Suržyk (“Neo-Suržyk”) during the period of Ukrainian independence after 1991 (cf. e.g., Flier, 2008; Hentschel & Reuther, 2020). This does not mean that in these three oblasts one has to expect only Neo-Suržyk. There is only a higher probability that a Russian based-mixed speech occurs in regions where Russian has been historically strong in Ukraine. In the central region, whose oblasts largely fall within the traditional Ukrainian dialect area, the “canonical”, Ukrainian-based Suržyk predominated almost universally, with only a few exceptions (respondents), e.g., in Xarkiv where Russian has been traditionally strong, too.

The “old” Suržyk developed over many decades, at the latest since the late 19th century (Hentschel & Taranenko, 2021). Due to these differences in potential development duration, it is at least doubtful that a Russian-based “Neo-Suržyk” emerged as a relative stabilised subvariety, as a little more than 30 years is most probably too short a time period for such a development. Anglo-Saxon dialect research (e.g., Trudgill, 1986)^5^ assumes three to four generations are necessary for the stabilisation of a new local or social dialect. One has to keep in mind the following: After 1990 the most important change in independent Ukraine regarding the linguistic situation has been the legal and factual promotion of Ukrainian by the government, mainly in the public sphere, in public institutions including educational ones. This (and perhaps the Russian aggression starting in 2014) may have been a stimulation for some individuals and families to shift from Russian to Ukrainian as the main code of communication or at least to increase their use of Ukrainian (cf. Verbytska et al., 2023). However, there were no other major changes in the daily surroundings of Ukrainian citizens. In contrast to Neo-Suržyk, the old, Ukrainian-based Suržyk had plenty of time to develop. An average social and professional career was unthinkable without Russian in the Russian Empire and the Soviet Union, and migration from other Russian speaking parts of the Soviet Union was – as is well-known – massive, of course with regional differences. One of the hypotheses in the above-mentioned project on the Black Sea Coast is that the difference between a Ukrainian- and a Russian-based Suržyk is possibly much less strictly clear cut but rather gradual and transitional. This assumption is based on the findings in Hentschel and Taranenko (2021), who report a far-reaching bilingualism on the level of individuals, of course with asymmetries, based on socio-biographic and/or regional differences.

Hentschel and Palinska (2022) recently referred to regional differences in Suržyk, which they propose to conceive as a mesolect between Ukrainian and Russian standard language on the one hand and autochthonous rural dialects on the other. They argue further that (at least) Ukrainian-based Suržyk should be seen as a mesolectal continuum, with far fewer regional distinctions than in the old autochthonous Ukrainian dialect continuum. This is why a clarification of regional differences and similarities is one of the main topics of this study.

Regarding the methodological structure of the corpus, it is relevant to note that the sub-corpora for the central region and the south stem to about equal parts from recordings of family conversations on the one hand, and from open (semi-structured) interviews on the other hand. The interviews topics covered questions relating to the linguistic practice of using Ukrainian, Russian and Suržyk, including attitudes, aspects of identity (ethnic, regional, religious), a Ukrainian, Russian or possibly Soviet orientation; with a special focus on language biography in the project on the Black Sea Coast. In both regions, Centre and Black Sea Coast, linguistic data were not collected in metropoles, due to the widespread opinion that these are widely Russian speaking locations, where Suržyk is hard to detect. There were only four metropoles: Kyïv, Xarkiv, Dnipro in the Centre and Odesa in the south. Of course, the surrounding oblasts were considered. In the project on the South, contrary to that on the Centre, villages were considered as well. There is no old Ukrainian (nor Russian) dialectal base in the South, due to the fact that a comprehensive Ukrainian and Russian colonisation started only in the 19th century, from different parts of Ukraine and Russia. In the Centre on the other hand there is an old autochthonous dialectal base in rural areas and traditional dialects are still in use, though most probably influenced by Russian as well.^6^ The linguistic landscape in the rural South is different, as migrants from other parts of Ukraine (and Russia) brought different dialects and regional vernaculars with them. Thus, as to levelling processes, villages here are in this respect rather comparable with smaller towns and so-called town-like settlements in the Centre.

The family conversations are cases of spontaneous intrafamilial speech among family members or also with randomly present friends, acquaintances and neighbours. The respondents were aware of the recordings, i.e., of a possibly constantly running recording device in a relevant room of their apartment for several days. Only selected portions of these recordings were evaluated, namely fragments with longer coherent conversation passages in mixed speech. The recordings of the open interviews, which lasted between thirty and ninety minutes, feature fragments of informal, although partially prompted speech.^7^ Often, the initial phases of the recordings, when some respondents did not yet use informal speech, were not evaluated.

Of the total material in the corpus outlined quantitatively above, only those sentences or utterances that can be described as hybrid were considered in the structural analyses. Regarding the question of a possible stabilisation of a form of mixed speech, of course only intrasentential code-mixing is relevant, and not alternating intersentential or interphrasal code-switching. Hybrid sentences can be seen as the core of a mixed variety like Suržyk. Spoken language corpora contain both complete and well-formed sentences as well as incomplete and elliptical utterances. These incomplete utterances as a rule still convey partial sentential meaning, allowing us to speak of intrasentential code-mixing.

Each word form in the corpus was described as Ukrainian, Russian, hybrid, or common. The procedure has been described in Hentschel et al. (2014) for Trasjanka; the same procedure was followed for Suržyk, with “Ukrainian” instead of “Belarusian”. A sentence, more precisely an utterance, is hybrid if it contains at least one Ukrainian and one Russian word form or at least one hybrid. One-word utterances were considered when the two-way context is hybrid.

Phonetic (accentual) characteristics do not play a role in the classification (cf. 3.1 below). In general, the probability of a hybrid constellation increases with the length of the sentence or utterance.^8^ The average utterance length in family conversations is about 6.3 word forms, in interviews about 7.5. This is not surprising due to the relatively few hypotactic constructions in speech.

If the total extent of the corpus was 730,000 word forms, about 530,000 of them are found in hybrid utterances, again with a proportion of slightly less than half for the central region and slightly more than half in the south.

## The Suržyk lexicon – usage frequency of Ukrainian and Russian lexemes

### Methodological remarks

The extent to which a highly mixed code with a considerable degree of variation is lexically shaped by the two (possibly more) donor codes can be measured by the usage frequency of word forms. We do not consider word forms as such, but sets of translation-equivalent lexemes of Ukrainian and Russian origin. In any case, it is necessary to abstract from inflection-morphological differentiations between the word forms. Therefore, (at least) one Ukrainian and one Russian lexeme were compared. The word forms in the corpus were lemmatised accordingly.

It should be noted that word forms of inflected parts of speech can sometimes only be assigned to a Ukrainian or Russian lexeme in certain inflection-morphological constellations (e.g., certain case and number constellations for nouns), but not in others; for example, (i) Ukrainian *kit* vs. Russian *kot* ‘cat’ in the nominative singular, but both ukr./russ. *kota* in the genitive singular, or (ii) both ukr./russ. *selo* ‘(larger) village’ in the nominative singular, but ukr. *sil* vs. russ. *sël* [s’ɔł] in the genitive plural. This problem only concerns Ukrainian and Russian lexemes that etymologically stem from a common root. The forms that are considered equal in the above examples (at least in the standard pronunciations) could show finer phonetic differences, most clearly in the pretonic /o/ in the first syllable of *kota*, where in Ukrainian an [ɔ] and in Russian a [ʌ] would be articulated in the standard pronunciation. However, differences such as the distinction here between akanje and okanje are evaluated as irrelevant for the fundamental specification as Ukrainian or Russian because Ukrainians also display this phonetic phenomenon of okanje in their Russian speech, which apart from phonetics is undoubtedly Russian (cf. Zeller 2022).^9^ As has been indicated above, such phenomena would be symptoms of a “superficial” Ukrainian accent in Russian, but not of a form of code-mixing.

For this lexical investigation, classifications were also determined by abstracting from morphonological alternations. The word for ‘language’ is *mova* in Ukrainian and *jazyk* in Russian. In the corpus, forms like *jazyci*, locative forms of the singular in Russian, are documented; in Russian, it would be *jazyke*. However, as the inflected form was clearly constructed from the Russian stem,^10^ it was assigned to the Russian lexeme. Morphonologically, it would be a hybrid.

### Lexeme-specific analysis: competition between Ukrainian and Russian lexemes

Intersentential mixing of two codes can be spontaneous (“real mixing”) or conventionalised (rather “mixture”). As indicated above, the prerequisite for conventionalisation phenomena, i.e., the emergence of a “fused lect”, is long-lasting, intense language contact spanning multiple generations, as well as the practice of mixed speech within the family circle as a central bridge between generations. As mentioned earlier, this condition is clearly met for a Ukrainian-based Suržyk. The transition from spontaneous mixing of two codes to their fusion into something third is fluid (cf. Auer, 1999). Notably, some structural variants (phenomena) may have already become stabilised with regard to the usually two values (expressions) of these variables from one or the other donor language in the sense that one value has generally become established, while the other still appears rather sporadically and spontaneously. Conventionalised and spontaneous mixing can overlap.

On the other hand, various scholars of Ukrainian (as well as Belarusian) linguistics still maintain the opinion that the variation of Ukrainian (Belarusian) and Russian elements in Suržyk (in Trasjanka) is spontaneous and chaotic.^11^

Conventionalisation involves the reduction of free variation in (at least roughly) synonymous or functionally equivalent elements (expressions, constructions, categories, etc.). It is not expected that this reduction reaches zero as long as the donor codes (or at least one of them) continue to be in use in the society where a fused lect develops. This is certainly the case in Ukraine, even though the use of Russian has declined after Ukraine’s independence and due to Russia’s occupation of the Crimea and aggression in the Donbass region.^12^

A reduction in the free variation between equivalent elements from two codes, here Ukrainian and Russian, can be demonstrated by analysing the usage frequency of the “competing” elements. If a Ukrainian or Russian element predominates strongly across the linguistic area under study, one can generally assume that the quantitative dominance of one element has become established, or there is at least a strong tendency in this direction. Furthermore, in such a large survey area as the one presented here, which includes subregions with quite distinct (linguistic) histories and dialectal differences, regional preferences must be taken into account, as shown recently for morphology and morphosyntax by Hentschel and Palinska (Hentschel & Palinska, 2022; Palinska & Hentschel, 2022). Isoglosses of dialectal distribution can come into play here. In other words, different regions may show different preferences, at least for some structural variables. For autochthonous dialects, dialect maps of word-semantic variables sometimes exhibit very diverse distributions of dialectal values (words), for instance the different terms for ‘rooster’ (AUM^13^). Such strong differentiations between often very small areas, however, are not to be expected in mixed subvarieties like Suržyk, which are shaped by social and language contact-related factors. Furthermore, the degree of mobility in today’s societies is much higher than at the time of the development of rural, peasant varieties. Yet, like traditional dialects, varieties of Suržyk typically serve for communication in the immediate environment, i.e., with acquaintances (family, colleagues, neighbours, etc.), which can promote locally or regionally limited stabilisations. For communicating with strangers, the use of standard languages (here Ukrainian and/or Russian) is relevant in both today’s society and the recent past. Speakers who regularly and frequently use the mixed code of Suržyk usually have an at least acceptable if not very good command of one of the donor languages in its standard forms (Hentschel & Zeller, 2017). Functional, intersentential code-switching is not uncommon between Ukrainian and Russian (at least before February 2022). Intersentential switching can also involve a switch to Suržyk, usually from Ukrainian, where functionality often exhibits features of style shifting (cf. Schilling-Estes, 2002; Chambers, 2002). Intrasentential code-mixing in Suržyk, i.e., within mixed utterances (sentences), is almost exclusively non-functional, however. Clear instances of conscious, functionally conditioned switches, say from casual Suržyk to “cultural” Ukrainian (or in the opposite direction), between two partial sentences within a mixed, complex one (interclausal alternations) or between two phrases of one sentence (interphrasal alternations), are very infrequent.^14^

Corpus-based studies on lexicons have certain limitations. Instances of the occurrence of competing elements must be represented with sufficient frequency to obtain reliable results (cf. Müller-Spitzer et al., 2018). While a corpus like the one available here, with its nearly three-quarter-million word forms, is generally large enough to be well-suited for comprehensive investigations of phonetic, morphological and morphosyntactic aspects (except for rare phenomena), this is not fully the case for lexical analyses.

In this study, configurations of competing Ukrainian and Russian lexemes, translatory equivalents, are called (interlingual) hyperlexemes. They are lexical variables with a Ukrainian and a Russian variant, and sometimes even multiple variants for each. Ukrainian and Russian aspect pairs, which are translation equivalents, were always combined into a hyperlexeme if they were based on a common root in their respective languages.

For the analysis presented here, only hyperlexemes that occur with a minimum frequency of 100 tokens were selected, yielding a total of 107 units.^15^ Just as in Zasorina’s (1977) frequency dictionary for Russian, which is based on a corpus of 1,000,000 word forms that only exceeds the one used here by about 250,000 word forms, most lemmas, i.e., hyperlexemes in this study, are attested only once. While the arbitrarily set minimum frequency of 100 allows relatively robust conclusions in the general analysis, the minimum limit for the subsequent comparative analyses in subregions of the survey area needs to be raised (see below).

Turning first to the general analysis of the whole survey area, Table 1 displays the results. The table is sorted by the proportion of Ukrainian variants or realisations of hyperlexemes (column “ukr. %”), in descending order. Hyperlexemes with the highest proportions of Ukrainian realisations are thus listed at the top. The leftmost column explicitly indicates the rank of the hyperlexemes (in descending order of Ukrainian realisations). Next to it are the hyperlexemes: Ukrainian realisation on the left, followed by “=” and the Russian realisation on the right. The next column displays the arithmetic mean (AM), which represents the average proportion of Ukrainian realisations of the hyperlexeme in different oblasts (regions).^16^ For example, if the hyperlexeme *tež* = *tože* (rank 104) has a Ukrainian realisation proportion of only 4.6%, then the Russian proportion is 95.4%. The column “$N$” to the right indicates the number of tokens for each hyperlexeme. (Just to repeat: All tokens in this column come from mixed sentences or utterances, so that they undoubtedly stand for the “core” of Suržyk.) The rightmost column represents the “preference class”, roughly mirroring the tendency of the given hyperlexeme towards a Ukrainian or Russian realisation. Note that the classification at this point of the analysis is an arbitrary one with borderlines at 90%, 80%, 66%, 33%, 20% and 10% of Ukrainian (and vice versa Russian) realisations. This grouping is illustrated by different colours.

**Table 1:**
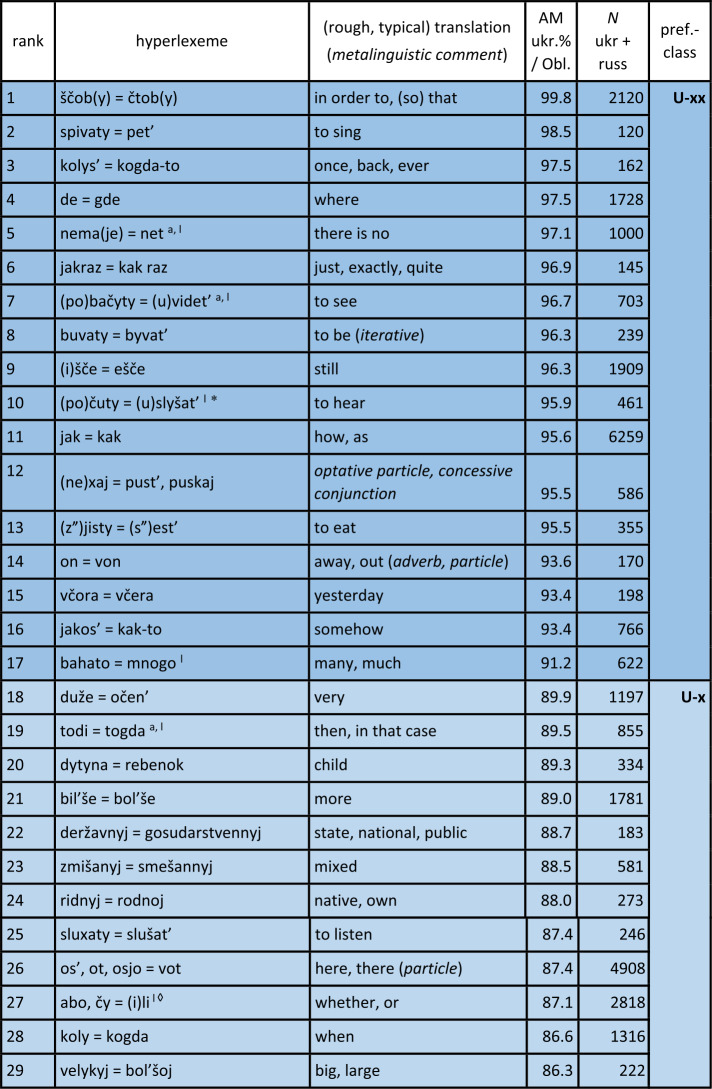

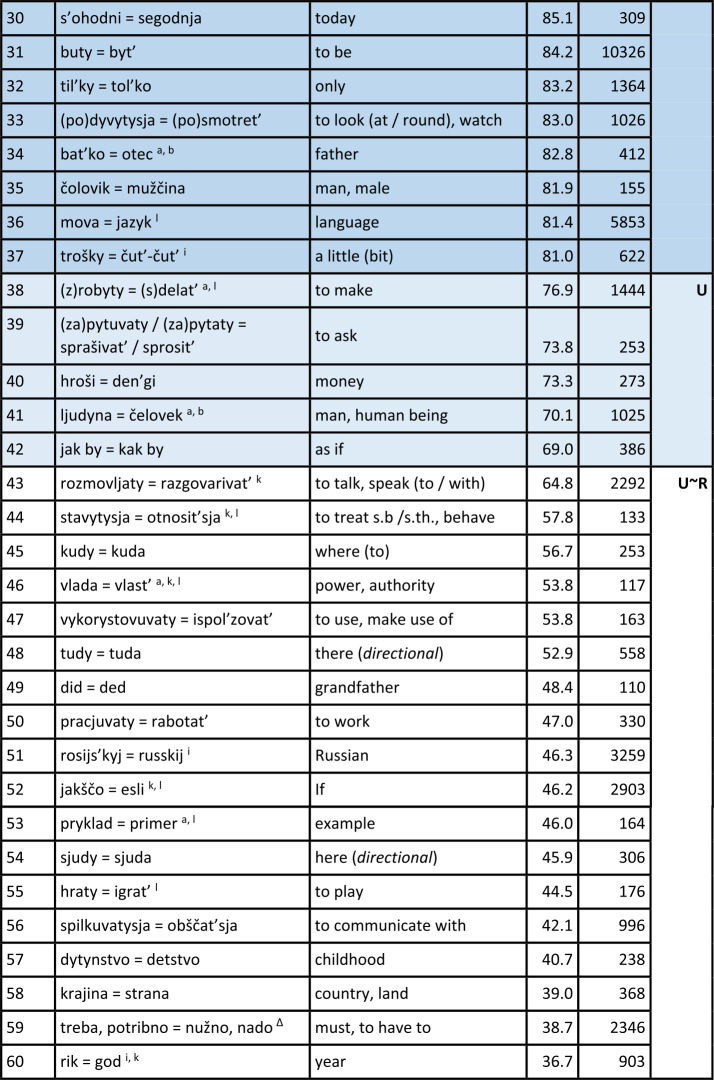

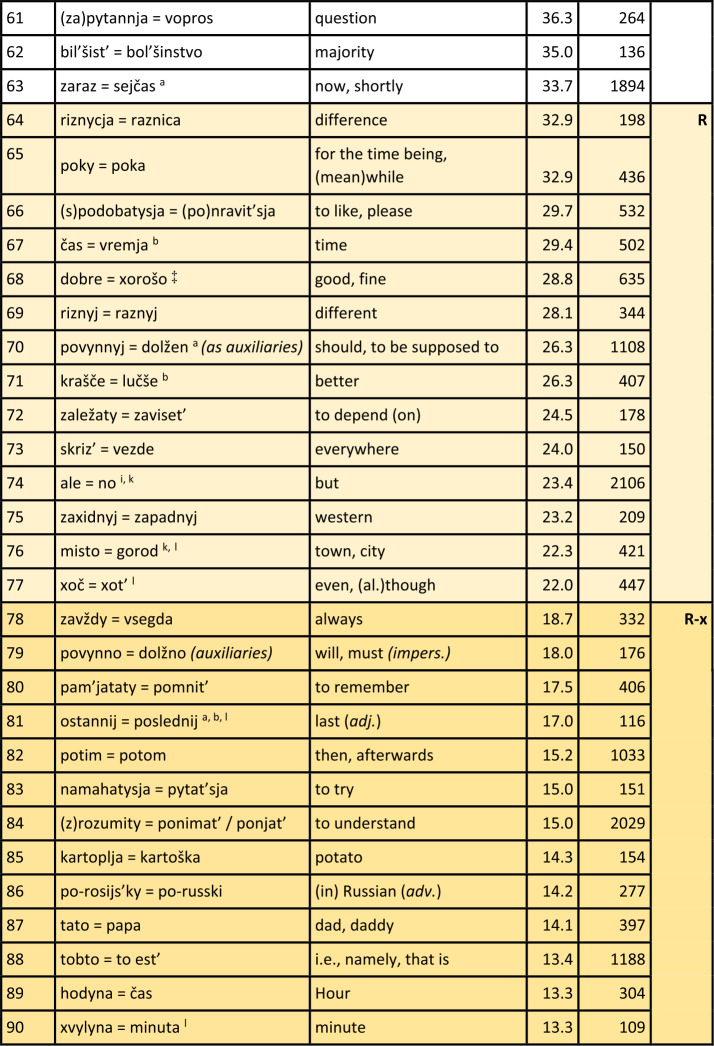

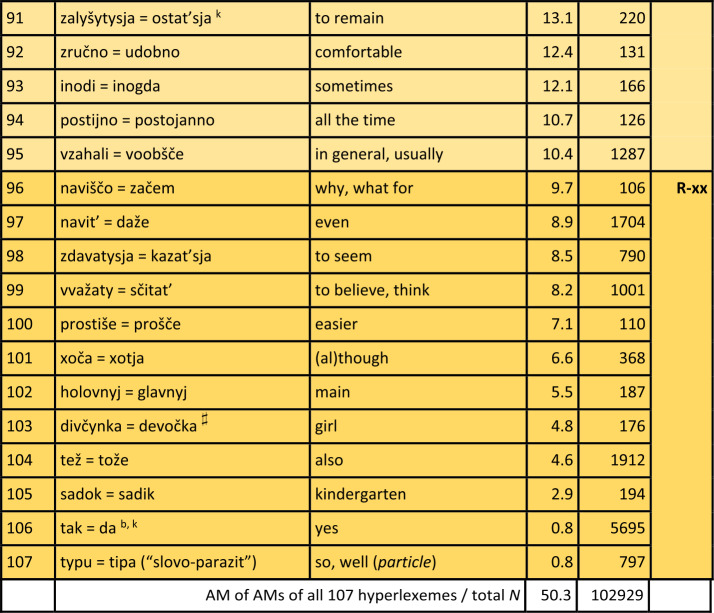
Ukrainian vs. Russian realisation of hyperlexemes (Color online)

^*^A similar phenomenon is found in the archaic ukr. *slyxaty* (SUM).

^⋄^A ukr. *ly* can be found in SUM-Hrin.

^△^*Treba, potribno = nužno, nado* (rank 59): Both ukr. *potribno* and russ. *nužno* are rare (2% and 1%, respectively). Russ. *nado* (72%) and ukr. *treba* (25%) dominate.

^‡^SUM refers to *xoroše*, accented on the first syllable. This expression appears six times in the corpus but was not included in the quantitative analysis.

^♯^SUM-Hrin and SUM feature a ukr. *divočka*, with stress on the first syllable like Russian *devočka*, both ‘little girl’. The former is not attested in the analysed corpus.

Notes on specific Ukrainian variants with a strong similarity to the Russian variants: a – western; b – central; i – colloquial; k – rare; l – archaic. (The information is based on SUM. More recent information is not available. Categories a) and b) are marked as “dialectal” in SUM. The specification “western” or “central” was determined based on sporadic information from dialectological literature. AUM provides no information on these lexemes.)

#### Comments on some additional elements

Before further discussing the findings in Table 1, comments on some potential hyperlexemes that were not included, even though they fulfil the quantitative criteria, will be presented. These considerations are at least of methodological relevance.

a) The first case is ukr. *maty* vs. russ. *imet*’ ‘have’ as expressions of possession (in a broad sense). There are about 250 examples of a corresponding hyperlexeme; the Ukrainian variant was realised in about four out of five cases. The possessor is indicated by a nominal phrase in the subject nominative. In contrast to West Slavic Polish, where this relation is almost consistently expressed by the corresponding verb *mieć* (etymologically related to the Ukrainian and Russian verbs), in Russian, the default expression involves a prepositional construction with the verb meaning ‘to be’ combined with the preposition $u$ plus a nominal phrase in the genitive to indicate the possessor. This construction is also common in Ukrainian (Kolečko, 1995; Popovyč, 2022). The regional distribution of these competing constructions or regional preferences are unclear. Phenomena of this type, where the competition is not purely lexical, cannot be discussed here, but require further, specific analysis.

b) Bilingual dictionaries usually give ukr. *xata* and russ. *izba* as translation equivalents, both referring to a hut, a peasant’s hut. However, in Russian, there is also *xata*, with a (roughly) corresponding denotation. The SSRJa (s.v.) explains that the latter carries a connotative nuance referring to objects in the western and southwestern parts of the Soviet Union or the Russian Empire (today Belarus, Ukraine, southwestern Russia). This does not exclude a humorous general use of russ. *xata* to refer to one’s own residence (BTSRJa s.v.). The simple translation equivalence from bilingual dictionaries is not suitable for our purposes. Nevertheless, in the corpus, there are about 200 instances of *xata* and none for *izba*.

c) The negating particles in response to yes-no questions (‘no’) and similar uses derived from such responses (e.g., *I don’t like things like this, no!*) are ukr. $ni$ and russ. *net*. There are about 3,600 tokens for a corresponding hyperlexeme in the corpus. However, the mentioned forms, which are also the standard forms, appear in only about 10 percent of cases each. Regarding russ. *net*, it should be noted that an older form *nit* exists in Ukrainian (SUM). However, this form, which appears in the corpus only three times, obviously does not contribute to the frequency of the phonetically similar *net* in Suržyk, where more than eight out of ten realisations are *nje*, which phonetically corresponds to russ. $ne$: [n’e]^17^ (see below). This *nje* / $ne$ or [n’e] cannot be unequivocally classified as Ukrainian or Russian at face value. As a negating reply, it is regionally present in Ukrainian only in the far west (LeksLviv s.v.). In Russian, the BASRJa (s.v.) describes $ne$ [n’e] in this function as “vulgar-colloquial” (“prostorečie”). In standard Russian, $ne$ [n’e] is a general sentence negator and marker of (contrastive) phrase negation. In standard Ukrainian, $ne$ [nɛ] serves as such a negator. The high frequency of *nje* / $ne$ [n’e] in Suržyk seems to be based on a process of its generalisation into a syntactic and reply negation marker, which neither conforms to the Ukrainian nor the Russian standard. On the other hand, the affirmative reply particle $da$, rather than the standard Ukrainian *tak*, is almost consistently present in the Suržyk corpus (rank 106), as in Russian. However, this $da$ also appears in central Ukrainian dialects. Nonetheless, since $da$ in Suržyk almost exclusively has this status, the limited dialectal presence of ukr. *da* might to a certain degree have facilitated the general adoption of *da* in Suržyk.^18^

d) The case pertaining to the term conveying ‘week’ is similar to b). In the standard languages, we have ukr. *tyžden’* and russ. *nedelja*, the latter being stressed on the second syllable, i.e., with a non-reduced, “clear” [e]. The Russian term for the week has a so-called “false friend” in Ukrainian, *nedilja*, designating Sunday. In this word too, the stress is on the second syllable. Although they only contrast in one segment (/i/ vs. /e/), the Ukrainian term for Sunday and the Russian term for the week, which contrasts with russ. *voskresen’e* ‘Sunday’, are perceptibly distinguishable. The latter hyperlexeme ‘Sunday’ is represented in the corpus only 45 times, with nine out of ten cases being the Ukrainian realisation *nedilja*. Nevertheless, in Suržyk a clear tendency towards using *nedilja* for ‘week’ can be observed. For this hyperlexeme with 179 instances in total, *nedilja* is observed in almost two-thirds of the cases, followed by the phonetically similar standard russ. *nedelja* in the first sixth, and the distinctly ukr. *tyžden’* in the second sixth. The homonymy between the term for the week and that for Sunday might seem functionally problematic at first glance. Nonetheless, the SUM lists *nedilja* as a colloquial variant for ‘week’ in Ukrainian, without further information on its regional distribution. Due to the small number of cases in the corpus, no further refinement is possible regarding regional or idiolectal differences.

e) The final example is ukr. *balakaty*, *kazaty*, *hovoroty* and russ. *govorit’*, all imperfective verbs with the meaning ‘to say, to speak (with / about)’. In certain contexts, all three Ukrainian verbs could certainly be translated by russ. *govorit’*. Ukr. *balakaty* is somewhat colloquial, more in the sense of ‘to chat’. In specific contexts, russ. *besedovat’* or the slightly negatively nuanced *boltat’* might be more appropriate translations. The interlingual equivalence relationships between these “verba dicendi” (verbs of saying) are complex, with a range of denotative and connotative nuances. Including them in the quantitative analyses of this study would not do justice to their complexity. It should be noted, however, that there are about 1,400 examples of ukr. *balakaty*, approximately 3,200 of ukr. *kazaty*, and around 2,200 of ukr. *hovoryty* and russ. *govorit’*, with more than half of these clearly being identifiable as Ukrainian, while the others show a certain degree of indistinctness. The predominant Ukrainian origin of the realisations of these “verba dicendi” is beyond doubt, especially since other, e.g., the mentioned Russian variants, occur extremely rarely. In general, lexical competitions with a complex relationship ($m$-to-$n$) between Ukrainian-Russian translation equivalents are not considered.

#### General discussion of the results

A total of 107 Ukrainian-Russian hyperlexemes were identified, for which nearly 103,000 word forms were recorded. This constitutes about 40% of all tokens in hybrid expressions (excluding pronouns and prepositions, as mentioned earlier), which can be classified as either Ukrainian or Russian.

Table 2 summarises the observations from Table 1.

**Table 2:** Quantitative Overview of the Results in Table 1

limit% ukr.	stable – variable	var.-cl.	pref.-cl.	*N* hl	% hl	*N* wf	% wf	*N* wf	% wf
> 90%	very stable	I	U-xx	17	16	17543	17	52324	51
> 80%	stable	II	U-x	20	19	34781	34		
> 66.6%	slightly variable	III	U	5	5	3381	3	28804	28
ca. 50%	(very) variable	IV	U ∼ R	21	20	17909	17		
> 33.3%	slightly variable	III	R	14	13	7514	7		
> 20%	stable	II	R-x	18	17	8761	9	21801	21
> 10	very stable	I	R-xx	12	11	13040	13		
				107	100	102929	100		

It can be observed that there is a large number of clear or at least relatively clear preferences either for a Ukrainian or for a Russian realisation of the hyperlexemes. Out of the recorded 107 hyperlexemes, 17 show a Ukrainian realisation in over 90% of the cases (darker shade of blue in Table 1). This group is referred to as preference class (pref. cl.) U-xx. Another 20 hyperlexemes are realised with Ukrainian equivalents in over 80% of the cases (medium blue highlighted – U-x). For these 37 hyperlexemes, there is persistent stability or even highly persistent stability of the Ukrainian variants of the hyperlexemes across the survey area. Furthermore, it should be noted that these two preference classes encompass more than half of the evaluated tokens, or word forms. Of course, both here and in the quantitative relations presented below the following applies: the higher the number of tokens for the hyperlexemes (column $N$ in the table), the more robust the findings are.

Similarly, the findings for hyperlexemes with a very high frequency of Russian equivalents are analogous: 12 of the hyperlexemes have a Russian realisation in more than 90% of the cases (darker ochre shade – R-xx), and another 18 have a Russian realisation in over 80% of cases (medium ochre – R-x). Thus, there are 30 hyperlexemes with a stable or very stable tendency towards the Russian variant. This accounts for an additional 21% of the recorded word forms.

Taken together, 67 of the 107 tested hyperlexemes exhibit very clear preferences, either for a Ukrainian or Russian realisation, making up almost three-quarters (72%) of the recorded word forms.

This leaves 40 hyperlexemes (clearly less than half of the 107) that show more pronounced or strong variation in the choice between a Ukrainian or Russian realisation. However, here it is also possible to identify units with somewhat more stable quantitative relations in both “directions”: 5 hyperlexemes show a proportion of over 66% Ukrainian realisations (light blue – U), 14 with over 66% Russian realisations (light ochre – R). There remain 21 hyperlexemes with a relatively balanced quantitative distribution between their respective Ukrainian and Russian units (white background – U ∼ R); this constitutes just under a fifth. Only a little more than a quarter of all word forms fall into this category of slightly or more variably realised hyperlexemes.

The threshold values (90, 80, or 66%) set for differentiating the classes above are, as has been pointed out above, arbitrary. Several hyperlexemes fall just above or below these values. The strength of the preference for a realisation corresponding to Ukrainian or Russian forms a continuum. This continuum will be considered in further analyses below.

Three central questions for the analysis were formulated above; a preliminary conclusion can be drawn for two of them:

(i) To what extent do the findings of the analysis indicate a stabilised mixture or spontaneous mixing? Or: Is the use of competing Ukrainian and Russian lexemes really chaotic, as many Ukrainian colleagues believe?

At least for the hyperlexemes of variation classes (var. cl.) I and II, i.e., preference classes U-xx / R-xx and U-x / R-x, which clearly tend towards a Ukrainian or Russian realisation across the board, the viewpoint of chaotic, unpredictable usage of competing Ukrainian and Russian lexemes can be rejected as absolutely misguided. Rather, a strong tendency towards reducing or restricting variation for these classes could be determined, which clearly encompass the majority of the hyperlexemes studied and the word forms available for them. If sporadic use of the usually less preferred Ukrainian or Russian lexemes occurs, that is to be expected: As mentioned above, as long as both donor languages of the mixed code are actively used in society, occasional deviations from the general preference can occur (cf. Auer, 1999). Especially for the other two variation classes III and IV, i.e., preference classes U and R as well as U ∼ R, the variation will be further investigated below in terms of regional differences.

(iii) To what extent is the Suržyk lexicon coined by Ukrainian or by Russian, even beyond the units analysed in more detail in this study?

In general, for the realisations of the 107 hyperlexemes, it can be observed that there is a balanced relationship between Ukrainian and Russian realisations in two respects. (a) There are similar numbers of hyperlexemes that show a stable or very stable tendency either towards a Ukrainian (U-xx / U-x) or towards a Russian (R-xx, R-x) realisation. (b) In Table 1, the last row not only indicates the total N of tokens but also the arithmetic mean of the arithmetic means of all hyperlexemes considered.^19^ Here, too, the Ukrainian and Russian shares are well balanced.^20^

### Regional differences

The central question (ii) concerned potential regional differences, which are to be ascertained by comparing the oblasts. It should be noted that the oblast boundaries are initially nothing more than a geographical coordinate system. Possible differences between the oblasts (or also between groups of oblasts with similar values) causally dependent on various conditions that shape the contact- and sociolinguistic landscape^21^ (see below).

Clear differences between the oblasts are, of course, improbable for the hyperlexemes in the two preference classes U-xx and R-xx, where either the Ukrainian or the Russian realisations of the hyperlexemes exceed 90%. Such differences were to be expected most frequently for hyperlexemes of preference class U ∼ R, where, in general, there is an approximately balanced ratio between the Ukrainian and Russian realisations. But of course, alternatively, such a balanced ratio could not be ruled out for U ∼ R in all single oblasts. But the latter hypothesis is not supported by the figures.

Tables 3 and 4 provide the results.

**Table 3:** Arithmetic mean of Ukrainian realisations (left-hand side) and $N $ of word forms of hyperlexemes by preference classes (horizontal) and regions (vertical)

Preference class (values in %)							OBL.	Preference class (*N*)						
U-xx	U-x	U	U ∼ R	R	R-x	R-xx		U-xx	U-x	U	U ∼ R	R	R-x	R-xx
97.8	96.3	99.6	72.8	71.6	66.2	13.5	Xmel	513	659	77	227	145	140	188
100.0	97.4	97.9	70.5	33.4	17.9	11.7	Čerk	841	1700	193	912	461	330	540
99.3	94.4	99.4	71.6	39.8	19.6	3.6	Vinn	388	799	58	382	247	177	421
95.7	88.9	72.2	48.5	33.5	20.4	4.8	Kyïv	712	1021	135	506	184	248	403
96.8	83.4	82.8	51.5	38.0	6.5	2.4	Kiro	523	1327	79	853	269	170	363
100.0	92.3	88.9	62.5	16.9	8.5	0.6	Žyto	386	829	120	376	215	142	208
99.8	93.8	89.1	51.3	26.5	9.7	3.6	Polt	1060	1775	204	889	396	371	545
90.3	75.8	68.0	35.2	21.1	8.1	10.1	Čern	692	1426	156	610	282	277	519
97.5	86.8	55.3	35.6	15.7	10.4	2.4	Sumy	584	1365	110	807	250	166	424
99.7	93.5	86.9	37.0	14.3	4.9	4.2	Dnip	1096	1957	159	1005	422	345	657
84.3	62.4	41.5	18.4	10.3	6.7	4.3	Odes	2902	6570	565	3110	1300	1824	2426
95.5	81.2	54.6	32.3	23.3	14.8	6.5	Xers	2964	5491	475	2881	1183	1488	2174
96.8	86.0	57.1	40.4	21.0	8.7	3.5	Xark	1301	2489	180	1297	427	754	821
91.5	72.8	35.6	28.7	19.2	10.5	6.6	Myko	3581	7373	870	4054	1733	2329	3351
							total:	17543	34781	3381	17909	7514	8761	13040

**Table 4:**
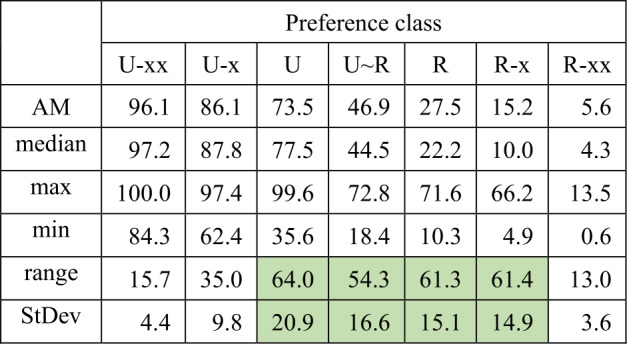
Arithmetic mean (AM), median, maximum, minimum, range (Max-Min) and standard deviation (StDev) of the proportions of Ukrainian realisations (according to Table 1) (Color online)

The data in Table 3 are ordered by oblasts based on a calculation of the strength of Ukrainian, Russian and Suržyk by (Hentschel & Taranenko, 2021, pp. 293–295, cf. esp. Map 3 and Figure 7 in their paper). The basis for the calculation were self-declared frequencies of usage of the three codes with more than 2,500 respondents, i.e., not linguistic data. Roughly, this order represents at least roughly the interrelation of the presence of Ukrainian and Russian on the axes from west to east and from central oblasts to peripheral ones, as illustrated in Hentschel and Taranenko’s Map 3.

As Table 3 illustrates, the general tendency of decreasing proportions of Ukrainian realisations that was found in the overall result is confirmed within the results of each oblast, i.e., it always decreases from left to right, with rare, punctual and insignificant deviations between two horizontally adjacent cells (cf. e.g., Čern R-x vs. R-xx or Xmel U-x vs. U). Some clear differences between the regions already emerge here: While the values for the (on the whole balanced) U ∼ R-class in Xmel’nyc’kyj (Xmel), Čerkasy (Čerk) and Vinnycja (Vinn) at the top of the list exhibit a Ukrainian proportion of 70% or more, the values for Odesa (Odes), Xerson (Xers), Xarkiv (Xark), and Mykolaïv (Myko) vary between 18% and 40%. Such differences correlate with Hentschel and Taranenko’s (2021) gradation. Regional differences will be further clarified below. The abundance of values in Table 3 is reduced in Table 4.

The median values, serving as a second measure of central tendency in addition to the AM, are generally less susceptible to individual “outliers” than the latter. The values of both are consistently very similar; therefore, outliers do not play a significant role.

As hypothesised, the “extreme” classes U-xx and R-xx show, if at all, only minimal differences between the oblasts. The relevant statistics here are range and standard deviation. As expected, they highlight the preference classes U, U ∼ R and R as particularly variable across regions, and even R-x (much less U-x). A further detail of these four classes is important: The values for range and standard deviation are comparable. This suggests at first glance that the (to remember, arbitrarily fixed) classes, esp. U, U ∼ R and R, could be united in a larger group with considerable regional variation. Class R-x is highly illustrative for the general problem behind the four similar values for range and standard deviation: Considering the values for the arithmetic mean and the median, this class can clearly be distinguished not only from R-xx on its right, but from R on its left as well. The fact that on the other hand R-x exhibits almost identical values for range and standard deviation with those of R is due to the relative share of Ukrainian in only one oblast: Xmel’nyc’kyj^22^ with 66.2% in R-x. The second highest share of Ukrainian in class R-x is much lower: 20.4% in Kyïv. In principle, the same is relevant for the classes U, U ∼ R and R. In spite of very similar values for range and standard deviation, they clearly differ in their measures of central tendency, arithmetic mean and median. The former circumstance is based on clear differences between a smaller subgroup of oblasts and a larger one. We must not forget that in the tables we are modelling a continuum (here of lexical preferences) by two arbitrarily scaled or subdivided dimensions: the seven classes and the oblasts. The latter are no more than a coordinate system for presenting regional differences of lexical preferences that by no means have to coincide with oblast borders.

The continuous character of these lexical preferences can be presented somewhat more illustratively by cartographic depictions, offering a clearer visualisation than an abundance of percentage and other numeric values can achieve. The individual values from Table 3 are repeated in the labels for the regions. The maps display the proportions of each preference class in the 14 oblasts: the saturation of the green colour indicates the individual values, with darker shades indicating higher Ukrainian proportions and lighter shades indicating higher Russian proportions. The modelling of the “extreme” preference classes U-xx and R-xx is omitted: no significant differences are present (cf. Table 3, left part). In the map for U-xx, all regions would appear as fully saturated green, whereas for R-xx, they would appear very pale green, almost white.

**Map 1:**
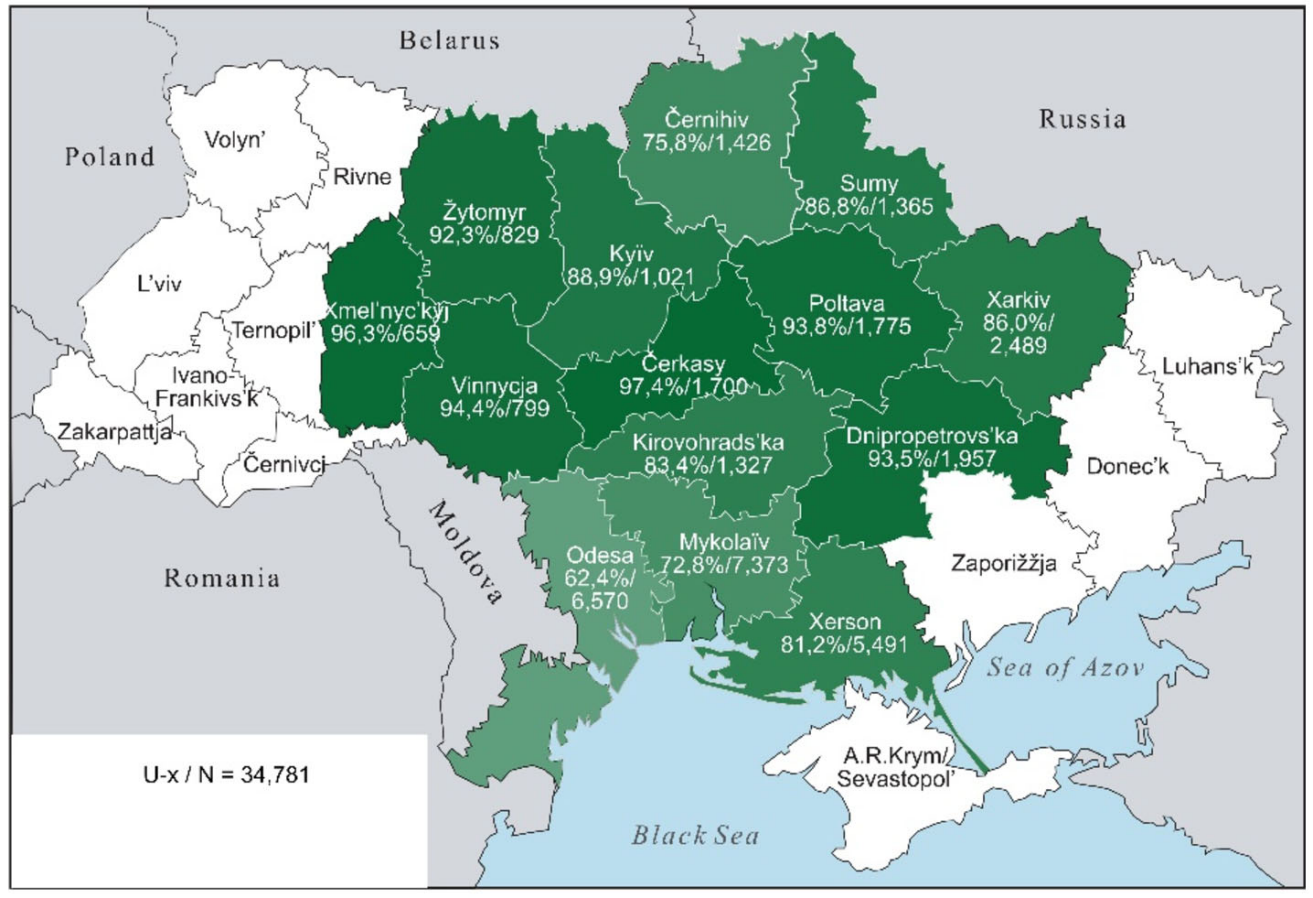
The preference class U-x in individual oblasts (Color figure online)

**Map 2:**
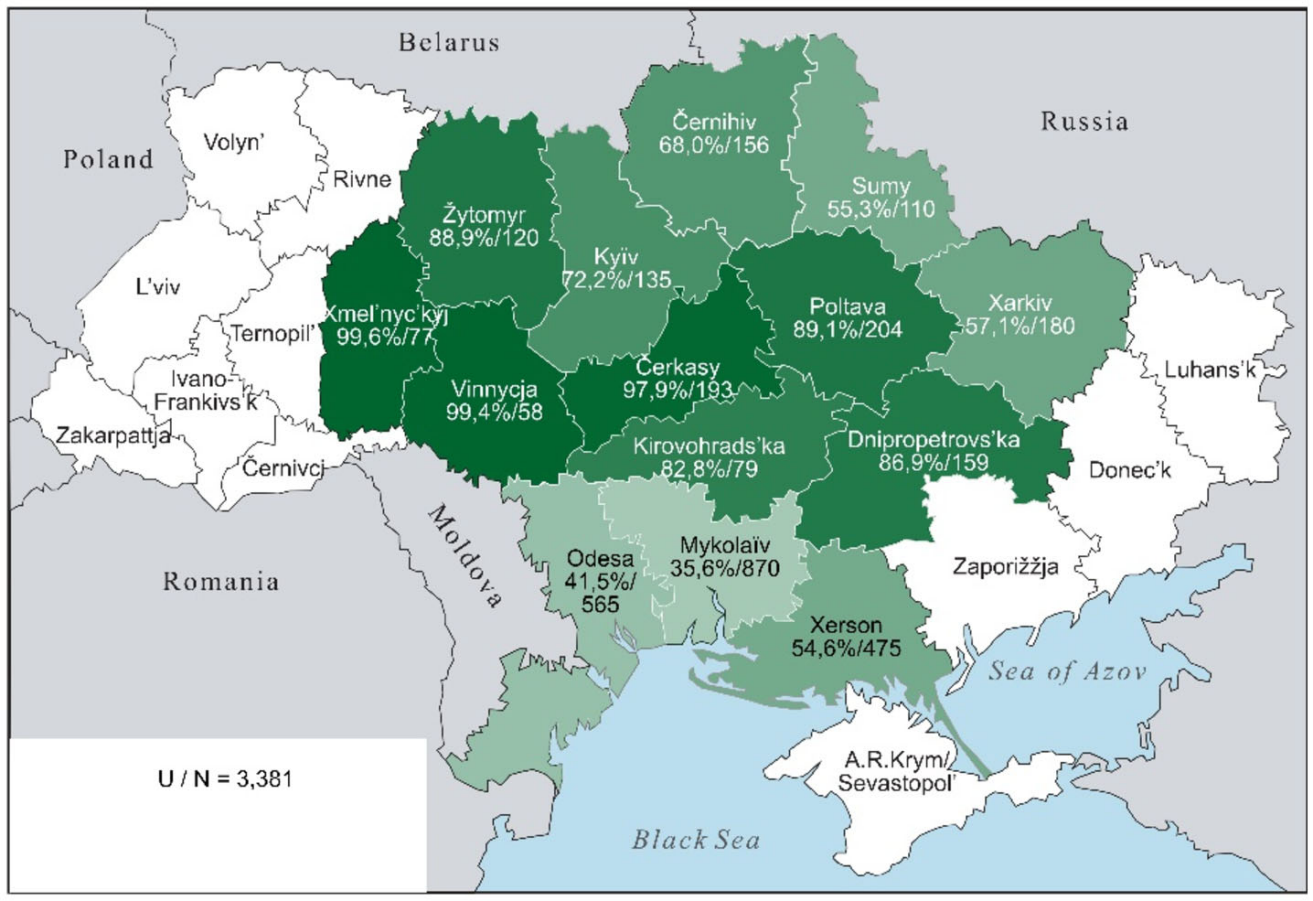
The preference class U in individual oblasts (Color figure online)

**Map 3:**
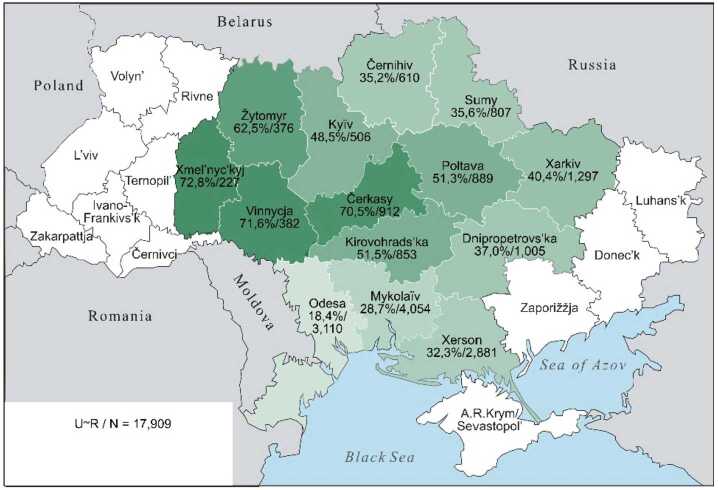
The preference class U ∼ R in individual oblasts (Color figure online)

**Map 4:**
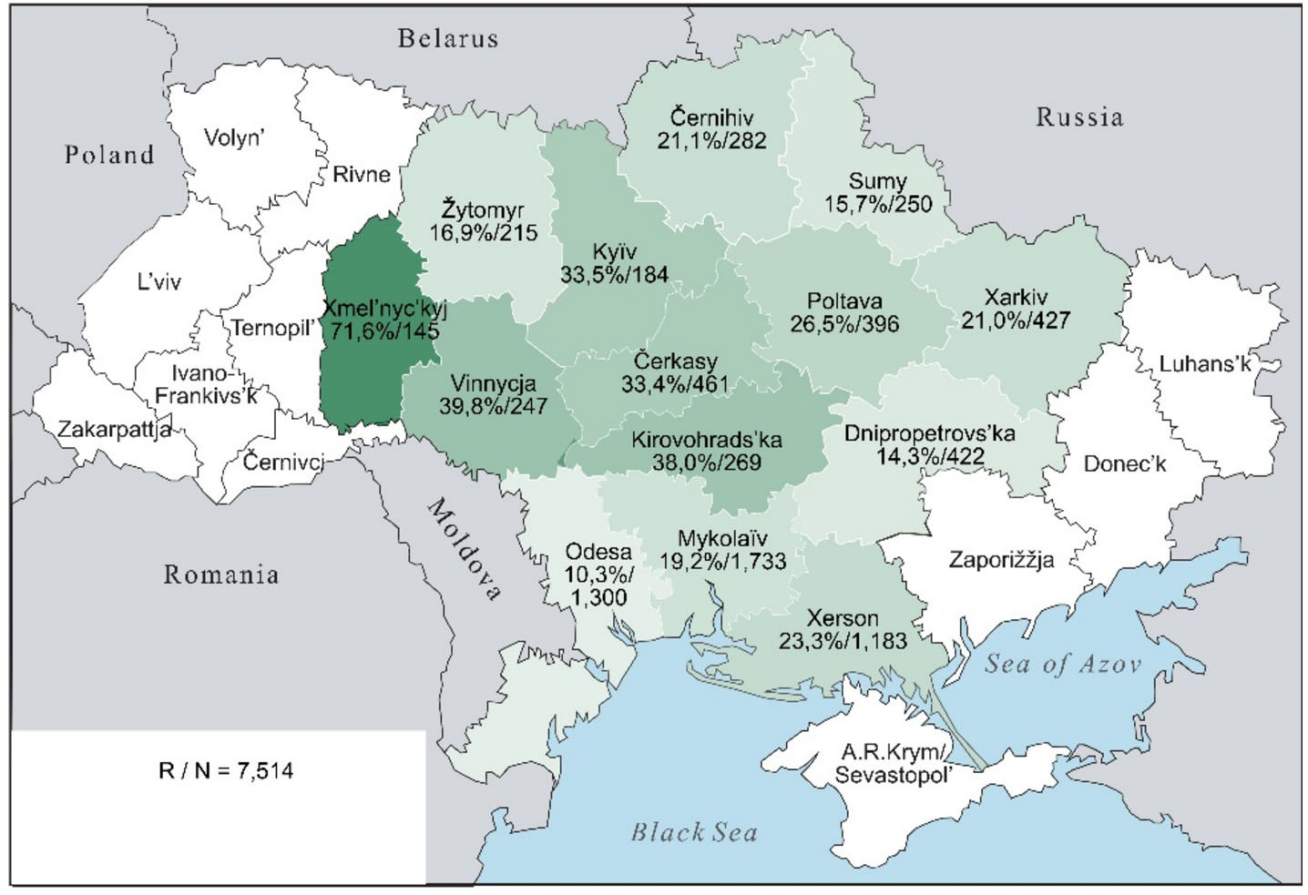
The preference class R in individual oblasts (Color figure online)

**Map 5:**
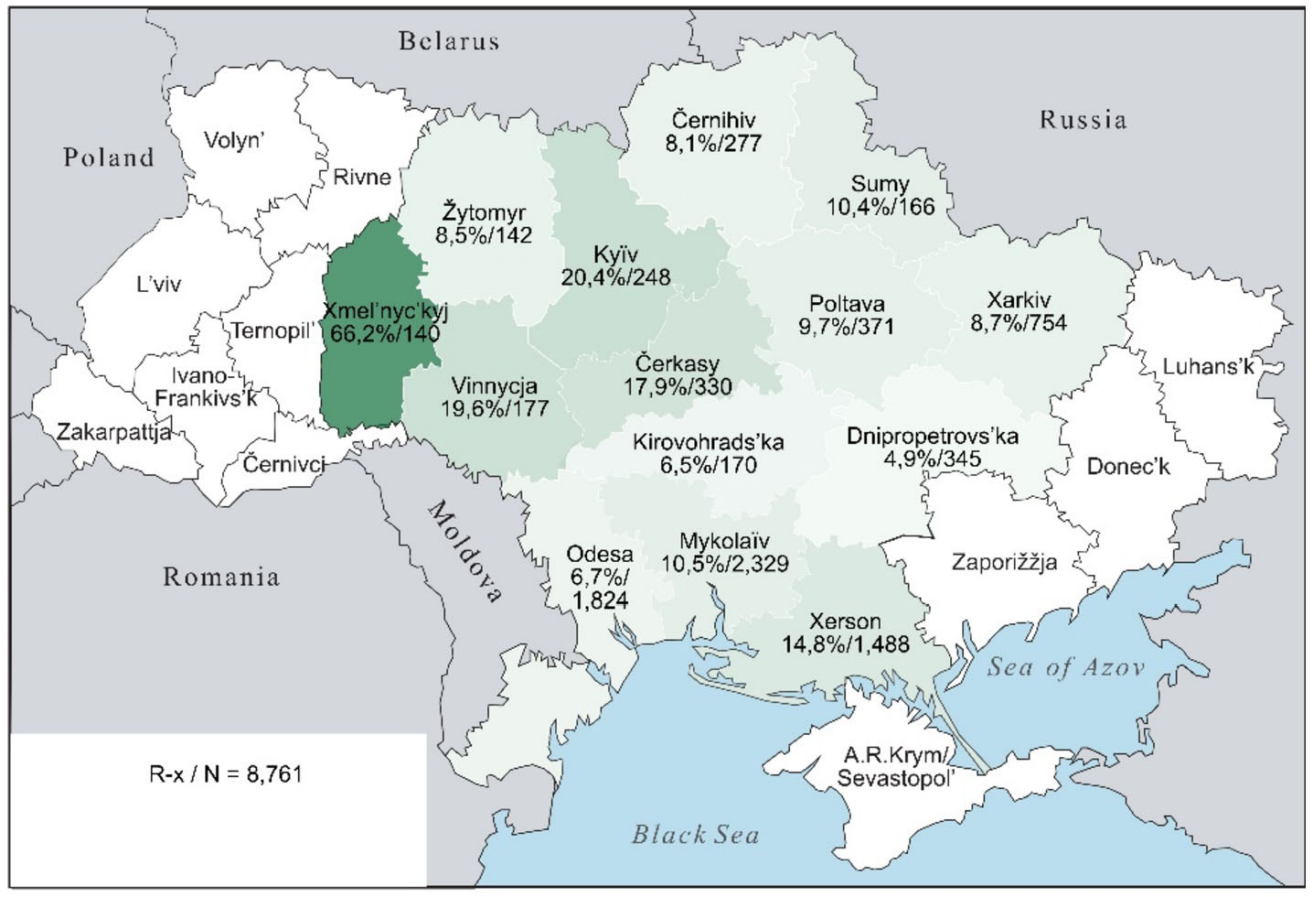
The preference class R-x in individual oblasts (Color figure online)

If in a map for preference class U-xx (not depicted) all regions would be a saturated dark green, Map 1, in contrast, shows a weakening of the green tone for U-x only in Černihiv on the border with Belarus and Russia, as well as along the Black Sea in Mykolaïv and Odesa, where the values for Ukrainian realisations of hyperlexemes fall below 80%. In Map 2, for class U, this trend intensifies: firstly, for four northern regions along the Belarusian and Russian border, starting from Kyïv to Kharkiv, and secondly, now in all three regions along the Black Sea. Map 3 for class U ∼ R then shows a wedge in the three western-central regions of Xmel’nyc’kyj, Vinnycja and Čerkasy, where values of over 70% still prevail. These three are spatially joined by an additional four regions – Žytomyr, Kyïv, Kirovohrad and Poltava, where values of around 50% or higher still exist. Around this central block, all the regions present significantly lower values. In Map 4, class R, the block dissolves, so to speak, from the edges, which further intensifies in Map 5 for class R-x. Only the westernmost Xmel’nyc’kyj still retains values around 70% in both cases. However, in a (unrealised) map for class R-xx, this region would also be shaded in a pale green with a value of about 14%, roughly similar to Xerson in Map 5. All other regions would then be depicted in even lighter shades.

**Graph 1:**
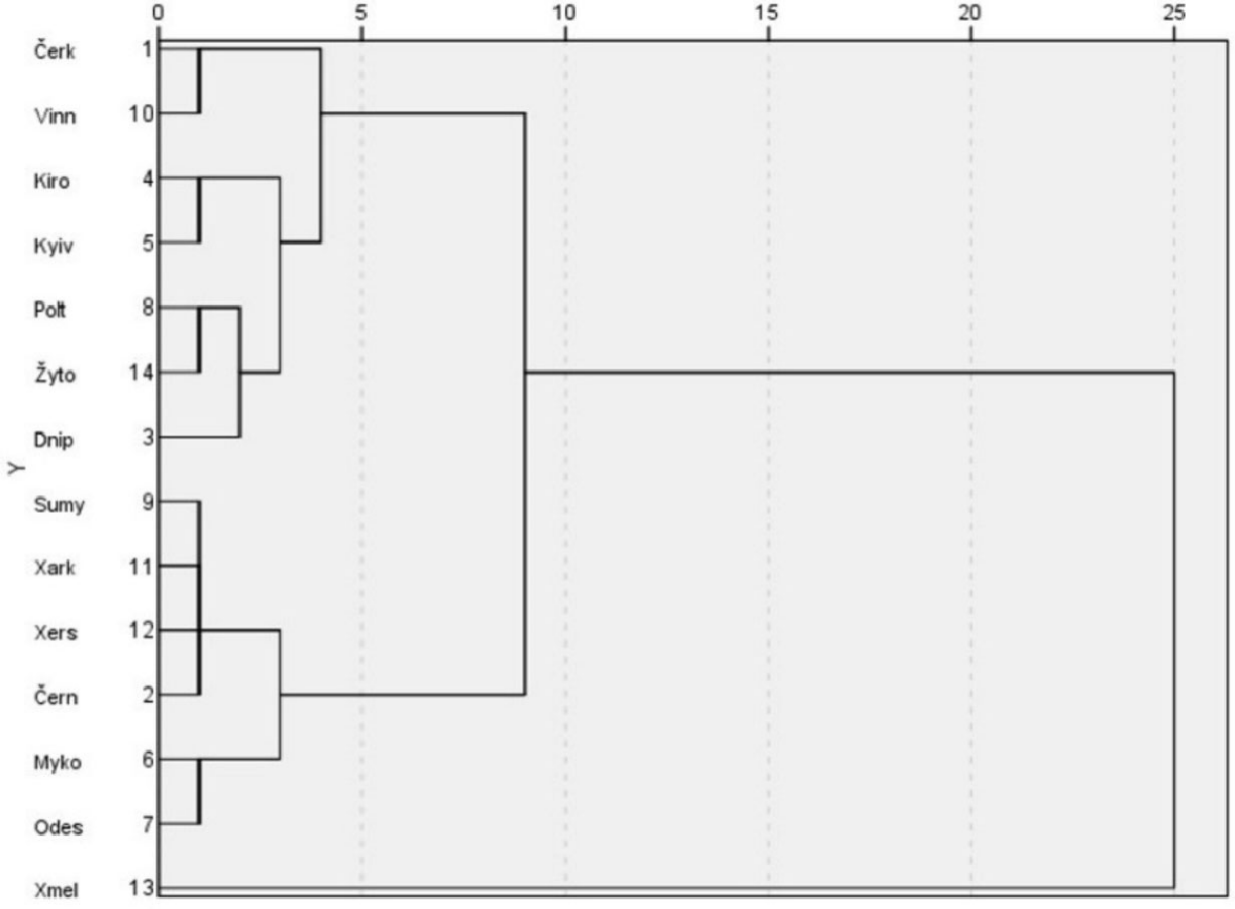
Dendrogram for cluster analysis of oblasts based on the values in Table 3 (left half) – combination of scaled distance clusters.

These intuitively presented tendencies can be statistically substantiated. The individual values for preference classes in the regions presented in Table 3 were subjected to a hierarchical cluster analysis (Bühl, 2019, pp. 635–651). The following dendrogram in Graph 1, illustrating the results graphically, depicts the differences (distances) between the regions based on their average values for the Ukrainian proportion in individual preference classes.^23^ The latter were postulated based on data from the entire survey area. However, only the classes that were found to be particularly variable among the regions in Table 4 were considered, whereas the extremely stable classes U-xx and R-xx were not included.

**Map 6:**
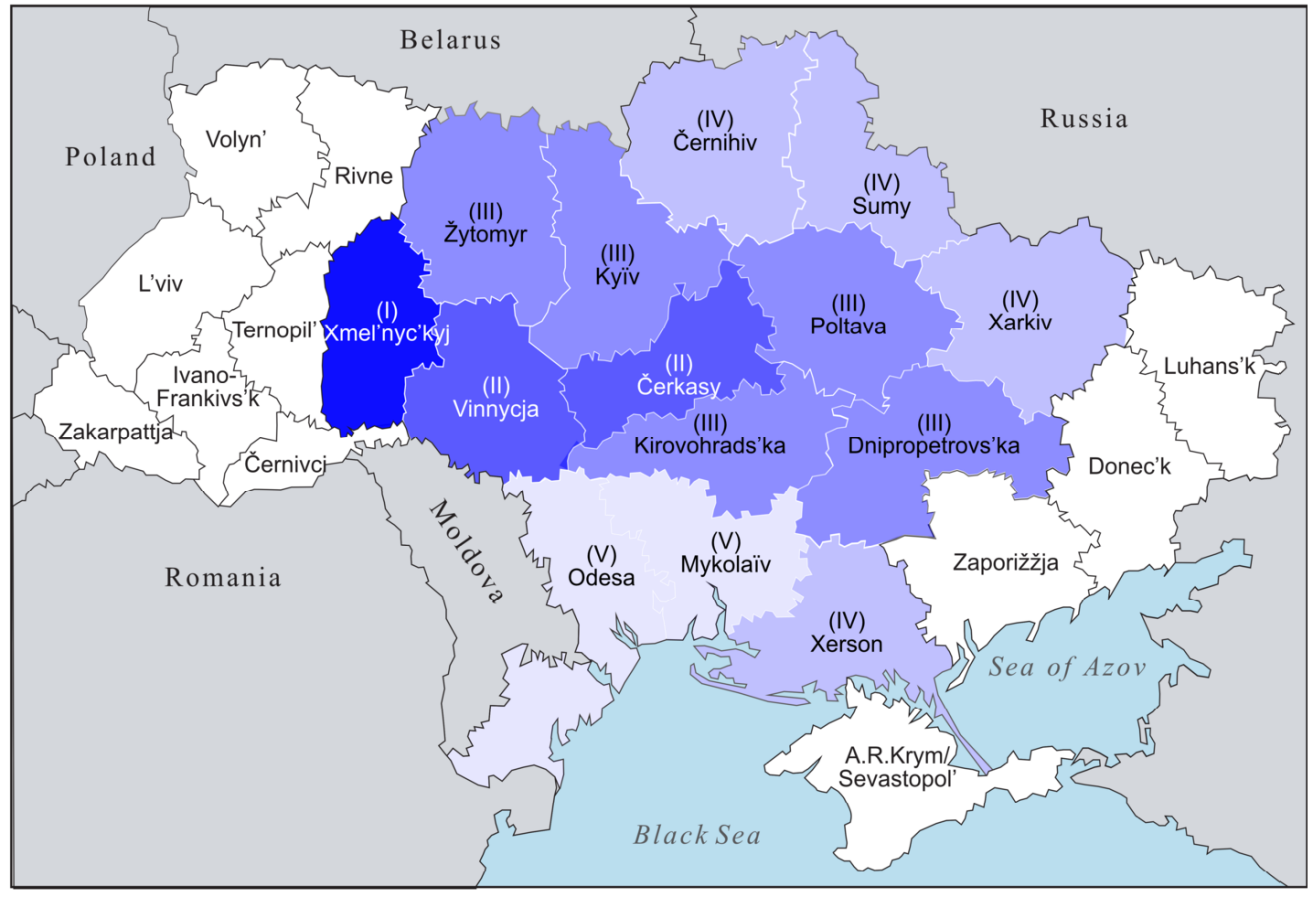
Cartographic illustration of the cluster analysis (Color figure online)

The dendrogram is to be interpreted as follows: (i) Xmel’nyc’kyj stands out very prominently from the other 13 oblasts, similar to Maps 4 and 5.^24^ (ii) The next split divides the 13 oblasts into two groups: the west-central group (Vinnycja, Čerkasy, Kirovohrad, Kyïv, Žytomyr, Poltava, Dnipropetrovs’ka) and a peripheral group at the border with the Russian Federation or the Black Sea, i.e., in the east or south (clockwise: Černihiv, Sumy, Xarkiv, Xerson, Mykolaïv, Odesa). (iii) Then, Vinnycja and Čerkasy in the west-central block stand out from the other five, as do Odesa and Mykolajïv from the other four in the eastern and southern peripheral block. This can also be illustrated cartographically (see Map 6).

The darker the blue shading of the oblasts, the stronger the Ukrainian realisation of hyperlexemes in the preference classes, indicating notable variation between Ukrainian and Russian realisations of the hyperlexemes.

The cluster analysis perfectly reflects (up to the third hierarchical level) the gradient of arithmetic means of the 14 oblasts in the five preference classes (Table 5).

**Table 5:**
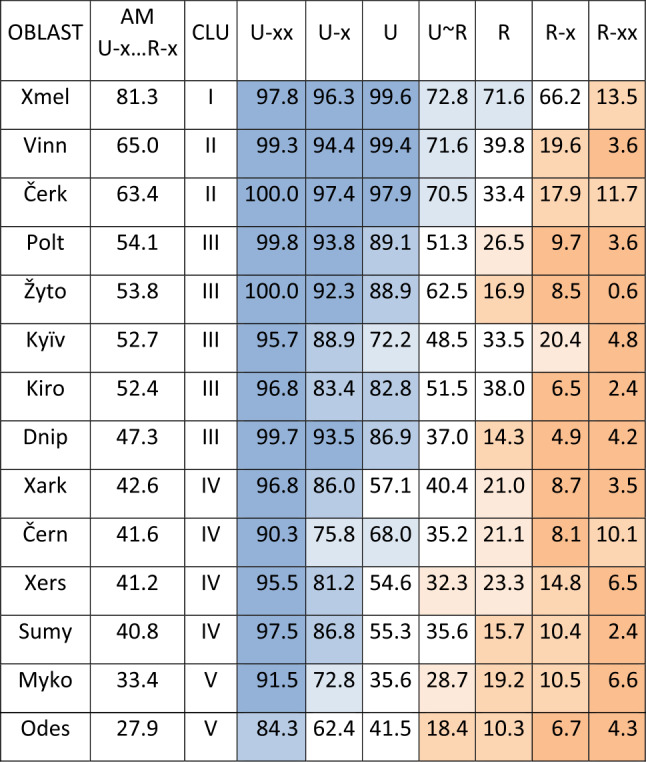
Cross tabulation of oblasts and preference classes sorted by the mean value of the preference classes in the oblasts (Color online)

The mean values (AM U-x ... R-x) from the five preference classes with a higher degree of variation between Ukrainian and Russian realisations of the hyperlexemes were taken from Table 3. When their values for all seven preference classes are shaded according to the thresholds as in Table 1, a “white diagonal” representing a region of stronger variation is visible from bottom left, U-x, in the Odesa oblast, where Ukrainian realisations generally show the lowest values, to top right, R-x, in the Xmel’nyc’kyj oblast, where Ukrainian realisations of hyperlexemes are most prevalent.

The analysis presented here, based on the usage frequency of lexical units and their depiction in geographical space, clearly shows similarities with the analysis of graded strength of Ukrainian usage in the same study area presented by Hentschel and Taranenko (2021), which is based on self-assessments of approximately 2,500 respondents.^25^

## On usage frequency, frequency effects and generalisation of results

This study included 107 hyperlexemes (types) in the analysis. Apart from the basic criterion that these are not “interlexemes” that do not show any formal differences between Ukrainian and Russian (aside from phonetic details), the criterion of a minimum occurrence of 100 usages in the total corpus was applied. Nevertheless, the 107 hyperlexemes analysed represent approximately 40% of all word forms (tokens of hyperlexemes) of the word classes considered (excluding pronouns and prepositions) that can be identified in hybrid sentences (utterances).

The fundamental question arises as to what inferences can be drawn from the analysis of hyperlexemes with a relatively high usage frequency of 100 or more tokens for those with lower frequencies. In this context, it is first important to note that the usage frequency of the 107 hyperlexemes analysed varies greatly. It ranges from the established minimum of 100 to about 10,000 (Table 6).

**Table 6:** Number of hyperlexemes by frequency levels

Token of hyperlexeme: at least …	*N* hyperlexeme	*N* hyperlexeme cumulative
5000	5	5
2000	9	14
1000	18	32
500	12	44
200	34	78
100	39	107

Table 3 has already shown that in both the clearly Ukrainian-influenced preference classes (U-xx, U-x) and the clearly Russian-influenced ones (R-xx, R-x), there are hyperlexemes that only slightly exceed the threshold of 100 occurrences set for the analysis (e.g., *spivaty* = *pet’*, rank 2, with ukr. %=98.5 / $N=120$ vs. *prostiše* = *prošče*, rank 100, with ukr. %=7.1 / $N=110$) as well as those with values well over 1,000 (e.g., *jak* = *kak*, rank 11, with ukr. %=95.6 / $N=6{,}259$ vs. *tak* = *da*, rank 106, with ukr. %=0.8 / $N=5{,}695$). The same applies to the hyperlexemes of the “intermediate” preference classes (U, U ∼ R, R). At face value, token frequency has no effect on whether hyperlexemes tend toward Ukrainian or Russian realisation or are highly variably realised. This informal impression of a lack of a correlation will be statistically tested.^26^

To test any potential relationship between usage frequency $N$ and the tendency toward Ukrainian or Russian realisation (measured by AM of AM Obl. Ukr.% in Table 1), the bivariate correlation was calculated with the correlation coefficient Kendall’s Tau^27^: r = 0.116 (sig. 2-sided 0.077). This confirms that among the 107 tested hyperlexemes, there is no correlation between usage frequency and Ukrainian or Russian realisation.^28^ Why some hyperlexemes strongly tend toward a Ukrainian or Russian realisation across the entire survey area, while others vary more or less strongly, obviously has nothing to do with usage frequency. Thus, there seems to be no reason to assume that this behaviour would be different for hyperlexemes with a token frequency of less than 100 in the corpus or those not contained in the corpus.

The reasons for the variation in the hyperlexemes, where it is observed, must lie elsewhere and will be investigated in subsequent analyses. The patterns of regional differences, as modelled in the maps, allow for hypotheses about the influence of sociobiographical factors. This includes, not least, the regionally varying presence of Ukrainian and Russian (as well as Suržyk), as described by Hentschel and Taranenko (2021). As Hentschel (2003) has shown for Belarusian, tendencies are often very lexeme-specific, which ultimately can only be illuminated through individual analyses, not just corpus linguistics.

## Summary and conclusion

Contrary to the widespread belief^29^ in Ukraine, Suržyk exhibits clear tendencies to reducing or restricting variation between linguistic elements that can be described as either Ukrainian or Russian. Here, the focus was on the lexicon. Among the competing Ukrainian-Russian lexical constellations referred to as “hyperlexemes” considered here, the majority of types and tokens (hyperlexemes and their word forms) show a clear tendency towards either Ukrainian or Russian expression. Importantly, a novel finding is that these fixations on either Ukrainian or Russian expression competitors are highly consistent across regions, yielding very similar outcomes for both central and southern Ukraine. This implies that the well-documented regional differences in the presence or use of Ukrainian and Russian standard languages in everyday communication do not play a significant role for these instances. Furthermore, this means for these pairs of Ukrainian-Russian translatory equivalents that no other factor plays any significant role in determining the occurrence of the Ukrainian-like or Russian-like expression in Suržyk, neither token frequency, which was tested, nor sociobiographical criteria such as age, education etc.

Especially the irrelevance of the possible factor of age for the choice of either the Ukrainian or the Russian expression of these hyperlexemes contradicts, in an apparent temporal perspective, the assumption that Suržyk can be considered an intermediate stage in a gradual language shift, to Russian until the 1980s, or to Ukrainian in independent Ukraine.

However, a second group^30^ of hyperlexemes also displays the fixation of a Ukrainian or Russian expression, but with regional differences. Furthermore, a third group of competing lexical constellations is observed, which exhibit variations between Ukrainian and Russian options in the whole area considered, with only weaker tendencies towards the preference of one over the other on a regional basis. For the latter two groups, where only partial, regional fixation or overall variation prevail, the factor of how extensively Ukrainian and Russian are used in each single region becomes relevant, thus, the criterion of different linguistic constellations in Ukraine in a historical perspective. The extent to which sociobiographical criteria play a role for these hyperlexemes has to be considered in a future study. It may turn out that these two groups can be modelled as one, variative group, with a graded “normative stabilisation” for individual hyperlexemes in different subregions.

The descriptive findings presented here are rooted in a consistent analytical focus on utterances that exhibit intrasentential code-mixing. The observed lexical fixations, the dominance of either Ukrainian or Russian elements and the displacement of the other, might appear surprising given the prevalent opinion in Ukrainian linguistics^31^ regarding disorder and chaos in such distributions. However, they align with the principle that complete denotative (and connotative) synonymy in languages is exceedingly rare, as explicitly described by linguist John Lyons (1968, p. 472) half a century ago. It is well known that languages and speakers tend to either abandon one expression or differentiate them denotatively or connotatively.

This study did not delve into the reasons why for some lexical competitions in Suržyk Ukrainian expressions are almost exclusively favoured while others exhibit a corresponding very strong preference for Russian expressions and still others (up to the present day) display a notable variation in the usage of Ukrainian and Russian expressions. Addressing this question comprehensively would require a series of individual studies, if feasible at all. This exploration could also involve investigating whether the Ukrainian and Russian expressions of the hyperlexemes that display significant degrees of variation carry finer denotative or connotative differentiations beyond the assumed translation equivalence. In this respect, the present study only examined whether the usage frequency, represented by the token frequency of hyperlexemes in the corpus, plays a role, which was negated.

In studies on morphological and morphosyntactic phenomena, Hentschel and Palinska (2022) and Palinska and Hentschel (2022) have illustrated regional differences in fixations of competing Ukrainian and Russian expressions. Suržyk should be seen as a continuum of mesolectal differentiations, akin to the concept of a dialect continuum, considering the numerous traditional dialectal isoglosses.

To be clear, Suržyk^32^ is a lect of its own, i.e. (here) with its own lexical norms, partially with regional differences. The identified widespread fixations of Ukrainian or Russian expressions of hyperlexemes indicate a cross-regional coherence, while the regionally varying fixations and preferences in other hyperlexemes suggest a geographically and thus cartographically measurable continuum. Classifying Suržyk as a lect of its own does not mean that it is a non-Ukrainian lect. Of course, the impact of Russian on its lexicon is considerable, but there is by no means a full Russian relexification. This has been outlined above. However, there are clear indications that the Russian impact on grammar is much weaker, but it exists here, too (cf. for example Hentschel, 2018, Del Gaudio, 2010, pp. 63–138). However, as long as we call English a Germanic language, in spite of a vast amount of non-Germanic lexical elements, it will be absolutely justified to call Suržyk a lect of Ukrainian: a mesolect continuum.

This study and the research projects on which this and other aforementioned studies are based, focus on the linguistic and sociolinguistic landscape of Ukraine between 2011 and 2021 in the centre of Ukraine and the Ukrainian Black Sea Coast. After February 2022 the linguistic situation in this and other areas of Ukraine is likely to undergo substantial changes due to the Russian invasion and the ongoing cruel war. One major reason is massive internal displacement and migration within Ukraine, as well as emigration abroad. These processes are likely to remain irreversible to a large degree, even if a favourable peace settlement is achieved for Ukraine.

Another reason lies in the altered or altering attitudes of Ukrainians towards languages or codes in their country. While studies like Hentschel and Zeller (2016) indicated that a clear majority of Ukrainians were at least neutral towards Russian, ongoing surveys by other researchers and media reports now show a noticeable emotional shift towards rejecting Russian and its usage among many Ukrainians. The attitude towards Suržyk was also relatively relaxed among the general population, while national-oriented elites stigmatised it. National cohesion, as Ukrainians at war impressively demonstrate, is a fundamental requirement for surviving this war. If the significance of Russian diminishes in a future free Ukraine, Suržyk will most probably change as well. It may become more Ukrainian, not only lexically. However, that it will rapidly disappear, as perhaps hoped for by those in Ukraine who, as Yuri Andruxovyč put it (without supporting this view himself), see it as the incestuous child of Ukrainian-Russian bilingualism, is more than unlikely for the coming decades.^33^ Currently, Suržyk seems to have a small advantage in that those who are “proficient in it”, i.e. who can easily express themselves in traditional Ukrainian-based Suržyk, are much less likely to be taken for infiltrators from the aggressor’s side.^34^

## Footnotes

1 Micro-areas (“lexical fields”) can indeed be very strictly structured, but they do not play a role in this study.

2 Originally both words denoted a mixture of good and bad ingredients, good and bad flour for bread of the poor (Suržyk) or hay as good cattle feed stretched with straw, when running out of feed (Trasjanka); cf. Taranenko (2007) and Cychun (1998).

3 For instance, there are only minimal differences in the grammatical categories. The means of expression differ to a considerable extent, often not in “substance”, but in distribution. This is evident, for example, in inflectional morphemes and prepositions.

4 The maps below illustrating the results of the analysis depict the surveyed area. Language data for Project DFG no. 155014374 were collected in 2010/11, for Project Fritz Thyssen Foundation no. 10.14.1.066 in 2014/2015, and for Project DFG no. 419468937 in combination with FWF no. I 4189-G30 in 2020/21. Since data collection was completed before the Russian Federation’s aggressive attack on Ukraine at the end of February 2022, these events could not yet influence the data and results.

5 In Anglo-Saxon dialectology, as in Trudgill (1986), the term “dialect” does not necessarily refer to a traditional subvariety rooted in the countryside, as in the German tradition of using the terms “Dialekt” and “Mundart”, but rather any subvariety that is established regionally, locally and / or socially (social dialect).

6 It is well-known that traditional dialectology, as it dominates (not only) in Ukraine, systematically ignores rather recent influences on the dialects from outside.

7 Further details about the sociobiographical backgrounds of the respondents, which are not considered in this study, are provided for the central Ukraine in Hentschel and Zeller (2016, 2017) and Zeller et al. (2019) and for the Black Sea Coast in Hentschel and Palinska (2022).

8 On the process for distinguishing individual utterances that do not constitute complete sentence structures in the corpus for Trasjanka, see Hentschel et al. (2014). A similar approach was used here.

9 As a matter of fact, it is well known that Russian speakers originating from northern Russia may also exhibit okanje, as it is a dialectal trait in those parts of the country.

10 The same root is found in Ukrainian “jazyk”, referring to the anatomical and culinary sense of “tongue”.

11 This is elaborated further in Hentschel (2017, pp. 27–29), which will not be reiterated here.

12 Presumably, the use of Russian is likely to further decline following the Russian Federation’s invasion of Ukraine, but since the data were collected prior to this event, they are not affected.

13 See AUM I Map 319; II – Map 331, III/1 Map 110, III/2 Map 117.

14 Cf. Tesch (2014, pp. 147–158) for the similar situation in the Belarusian-Russian mixed code of Trasjanka.

15 Pronouns and prepositions were not considered. The former are essentially referential units rather than lexical ones. The latter are often either grammatical markers or units governed by lexemes (often verbs) with which they are analysed. Lexical-paradigmatic oppositions mainly involve local or temporal uses of prepositions, with few substantial distributional differences between Ukrainian and Russian.

16 The arithmetic mean (AM) was not directly calculated based on all occurrences of each respective hyperlexeme in the entire survey area, but rather as the average of the 14 percentage shares of the Ukrainian realisation in each oblast. This approach prevents oblasts with substantially more data from having a stronger quantitative effect than those with more limited data. As mentioned above, a much larger database is available for the three oblasts in the Black Sea region.

17 The vowel can be pronounced more openly in Ukrainian and more closed in Russian.

18 Just as in Belarusian Trasjanka (Hentschel, 2013), the pattern of affirmative and negative particles is *da* – $ne$. However, unlike in Ukrainian, the Belarusian $ne$ is supported by the standard language. In contrast, *tak* is affirmative in both Ukrainian and Belarusian standards.

19 Calculating a total AM from the individual AMs of hyperlexemes ensures that extremely frequent hyperlexemes do not disproportionately influence the result compared to less frequent ones. However, calculating a general AM without this adjustment only yields slightly different values: 54% Ukrainian and correspondingly 46% Russian realisations of hyperlexemes. This suggests that token frequency does not significantly influence whether a Ukrainian or Russian expression is preferred for the corresponding meaning in Suržyk.

20 It should be noted in passing that this confirms that Suržyk is significantly less influenced by Russian than the Belarusian Trasjanka (cf. Hentschel, 2013) in the “semantic” lexicon, as had been suggested by Hentschel (2018) rather roughly.

21 The same holds, in case of an oblast-specific high degree of variation, for the situation within individual oblasts. However, our data does not allow for a differentiation of geographic areas smaller than the oblast.

22 The specificity of Xmel’nyc’kyj cannot be explained by an analysis of lexical data alone. It showed up in questionnaire-based surveys as well (cf. Hentschel & Taranenko, 2021). According to the latter study, the oblast Xmel’nyc’kyj is the one with the highest presence of Ukrainian in everyday life, compared to the other oblasts (the same there and here). In Hentschel and Palinska’s (2022) study on the distribution of Ukrainian and Russian infinitive endings in Suržyk, Xmel’nyc’kyj (though in this case together with neighbouring Vinnyc’ja) showed only the ending which is the default in Standard Ukrainian (-*ty*), where in all other oblast there was a clear dominance of the default ending in Standard Russian (-$t'$), which is shared by some (by far not all) Ukrainian further in the east. This means that similarities between the local or dialectal usage norms on the one hand and norms of the relevant two Standard languages on the other hand are another important factor. This must be analysed on broader, non-lexical material. Note, that for the majority of Ukrainian variants of the hyperlexemes no information on presence or absence in dialects is available.

23 In principle, such an analysis could also be performed for individual hyperlexemes. However, many hyperlexemes would have an insufficient number of occurrences in individual oblasts, which would distort the comparison. A coarser measure across groups of hyperlexemes, i.e., the preference classes, is preferred here.

24 As to the specificity of Xmel’nyc’kyj see fn. 22 above.

25 Compare especially the above Map 6 with Map 3 in (Hentschel & Taranenko, 2021, p. 294).

26 Genuine frequency effects, i.e., those that are independent of other factors such as social or regional factors (related to respondents) or lexeme-related (expressive or semantic) factors, are difficult to model. As Pfänder et al. (2013) noted, corpus analyses primarily reveal correlations (or the lack of them) between frequencies and other characteristics of relevant elements (in this case, Ukrainian or Russian realisations of hyperlexemes). This has been done here. A later study will further analyse potential correlations between usage frequency and social factors.

27 This is the relevant correlation coefficient because the values of both variables were identified as not normally distributed by the Kolmogorov-Smirnov test.

28 For readers less familiar with statistics, it should be noted that correlation coefficients can take values between 0 and 1. A weak or low correlation is considered to exist at a value of 0.2 (Bühl, 2019, p. 422), which is below the value observed here, not to mention the lack of significance.

29 See, e.g., Trub (2000, p. 54), Bracki (2009, p. 249), Masenko (2023, pp. 153–154). It should be noted that these viewpoints, which postulate a spontaneous, unpredictable, disordered and even chaotic occurrence of Ukrainian and Russian elements in mixed Suržyk speech, are “analytically” rooted more in informal observation and a holistic-impressionistic approach to mixed Ukrainian-Russian speech. Analytical methods (experimental or corpus-linguistic), as developed in the last half-century after William Labov’s early work in variation linguistics, are not taken cognisance of. The same applies to theoretical concepts like the differentiation between inter- and intrasentential code-switching or code-mixing when it comes to distinguishing Suržyk from Ukrainian and Russian speech.

30 Please note that the use of “group” here is informal. It must not be identified with the classes like U-xx, U-x etc., although the first group roughly corresponds to classes U-xx/-x and R-xx/-x, the second to U and R, and the third to U ∼ R. The classes were fixed arbitrarily for the ease of presentation of the shares of Ukrainian (and diametrically opposed Russian) realisations of the hyperlexemes in general, initially not considering regional differences. If we were to establish such a classification for subregions (down to oblasts), then many lexemes would belong to different classes in different regions. This is already indicated in Table 5.

31 Occasionally, corresponding expressions or the general attitude toward Suržyk are not devoid of national undertones, which can be perceived as anti-Russian or at least anti-colonialist. This is emotionally understandable, considering that the dominance of Russian has been accompanied by varying degrees of repression or restrictions against Ukrainian, particularly since the 19th century (cf. Danylenko & Naienko, 2019). The question is, whether such an ideological approach to Suržyk is helpful for academic insights. I think that it is not.

32 Note that here traditional, Ukrainian-based Suržyk is at issue. A systematic differentiation of this prototype Suržyk from a much younger Russian-based Neo-Suržyk was not an aim of this study, but will follow soon. Hypothetically, it can be assumed that such a Neo-Suržyk would (to certain degree) share the norms of the traditional variant, wherever Russian traits are established in the latter.

33 For the background to this “incestuous child”, see Stavyc’ka (2014) and Hentschel (2014).

34 This is based on information from one of our Ukrainian collaborators who, initially in the Xerson region, fell under Russian occupation until being liberated by Ukrainian forces. During the occupation, he supported his Ukrainian compatriots as a clergyman, including in practical matters.
